# Oxidative stability in meat (pectoralis major) of broiler orally supplemented with essential oils of allium sativum, Curcuma longa, Zingiber officinale, and Cinnamomum zeylanicum

**DOI:** 10.1093/tas/txae073

**Published:** 2024-04-29

**Authors:** Olatunji Abubakar Jimoh, Sule Bamidele Akinleye, Chigozie Joy Simon, Aderonke Opeyemi Kayode, Mary Olajumoke Akande, Tolulope Emmanuel Ogunjobi, Labeeb Taiwo Tijani, Kehinde Tosin Ayileye

**Affiliations:** Animal Production Technology Department, Federal Polytechnic Ado Ekiti, Ado Ekiti, Nigeria; Animal Science Department, University of Ibadan, Ibadan, Nigeria; Animal Production Technology Department, Federal Polytechnic Ado Ekiti, Ado Ekiti, Nigeria; Animal Production Technology Department, Federal Polytechnic Ado Ekiti, Ado Ekiti, Nigeria; Animal Production Technology Department, Federal Polytechnic Ado Ekiti, Ado Ekiti, Nigeria; Animal Production Technology Department, Federal Polytechnic Ado Ekiti, Ado Ekiti, Nigeria; Animal Production Technology Department, Federal Polytechnic Ado Ekiti, Ado Ekiti, Nigeria; Animal Production Technology Department, Federal Polytechnic Ado Ekiti, Ado Ekiti, Nigeria

**Keywords:** antioxidants, cinnamon, cooking loss, essential oil, lipid oxidation, pectoralis major

## Abstract

Lipid oxidation is a normal process in living muscles, but is escalated postmortem due to the loss of inherent antioxidant defense, which causes quality deterioration of meat. This study investigates the effects of essential oil (EO) supplementation to the drinking water of broiler chicken on physical properties, antioxidants, and lipid oxidation in *Pectoralis major* during frozen storage. Two hundred day-old chicks of arbo acre were allocated to five groups; control (T1) and the groups supplemented with: *Allium sativum* (T2)*, Curcuma longa* (T3)*, Zingiber officinale* (T4) and *Cinnamomum zeylanicum* (T5) at the level of 300ml/L into drinking water throughout a 49-d study. Thereafter, birds were slaughtered and breast meat excised for assessments during a 28-d storage period at 4 °C using standard procedure. The results show that cooking loss of *Pectoralis major* from T1 birds was not significantly (*P* > 0.05) different from that of T4, and were significantly higher than those of T2, T3, and T5 birds. Meat from T5 birds showed the lowest drip loss. The results for total antioxidant activity are not similar among sampling days. In general, control group showed inferior values, but T2 and T4 had greater values on days 0 and 28. The rate of lipid peroxidation increased with time; however, EOs administration markedly reduced the peroxidation rates compared to controls. The catalase activity of breast meat was significantly declined from day 14, but was enhanced as an effect of EO consumption especially in group T5 at 21 and 28 d. Supplementation of garlic, turmeric, and cinnamon EOs to broiler chickens increased glutathione peroxidase in breast meat on days 21 and 28, while turmeric EO enhanced superoxide dismutase up to 7 d. In conclusion, EOs are valuable supplements for broiler chickens and potent in enhancing meat quality and prolonging the shelf life.

## Teaser

An investigation on how essential oils in broiler chicken diets revolutionize meat quality and prolong shelf life, with garlic and turmeric extracts having higher antioxidant potency. This study unveils a breakthrough method for enhancing poultry products, ensuring freshness and quality for 28 d of frozen storage.

## Introduction

Chicken meat is susceptible to oxidative degradation owing to its high content of polyunsaturated fatty acids (PUFA; Branciari *et al*., 2017). Oxidative deterioration in meat is observed as nutrient and drip losses, discoloration, and limited shelf life (Contini *et al*., 2014). Oxidation in lipid and protein fractions of meat is the non-microbial cause of quality deterioration during processing, due to its easy susceptibility to oxidative damage owing to fast depletion of antioxidants post-mortem (Xiao *et al*., 2013). The animals’ ante mortem dietary intake greatly impacts the vulnerability of meat to postmortem peroxidation as demonstrated by Zhang et al. (2011), who showed that breast meat of domestic fowls fed with dietary oxidized oils recorded an increase in peroxidation of lipid and protein compared to those fed with antioxidant supplemented diets.

Lipid oxidation results in deterioration in the quality of meat and meat products by inducing off-flavor and lowering shelf life (Huang and Ahn, 2019). Lipid oxidation is the main source of meat structural degradation, begins antemortem in the muscles and is peaked after slaughter owing to environmental changes and the loss of inherent antioxidant defense (Branciari et al., 2017). Thiobarbituric acid reactive substances (TBARS) are a key quality indicator of peroxidation lipidation and values < 3 mg/kg are recommended for meat and meat products (Cadun et al. 2005; Ürüşan and Bölükbaşı, 2020).

Hence, growing efforts tend to be geared towards the enhancement of oxidative stability of chicken meat through the administration of various natural antioxidants to birds, leading to an increase in the market value of these products (Branciari et al., 2017). Antioxidant supplements can be administered via dietary interventions to eliminate the oxidative events in meat products (Branciari et al., 2017). However, in vivo, nutritional strategies are more efficient than in vitro postmortem inclusion of antioxidants in meat, because the antioxidant compounds are preferentially deposited where it is most needed (Symeon et al., 2014). Bioactive compounds of herbs have been incorporated into chicken diets to enhance meat quality via the alteration of fatty acid content (WHO, 2020).

The potency of different essential oil (EO)s to influence meat oxidative stability is demonstrated by Anjum et al. (2004). Antioxidative activities of chicken meat can be improved by herbal antioxidant supplements, like EOs, with potency to influence oxidative stability of tissues, as have been assessed on chicken-fed supplements of thymol, carvacrol, oregano, or rosemary oil (Hashemipour et al., 2013).

EOs (EO) are organic oily fluids derived from different parts of a plant, with various bioactive ingredients that can be used in animal nutrition (Bakkali et al., 2008), and have shown biological implications on the tissues, altering metabolism of fat. EOs are obtained by various methods including fermentation and extraction; but steam distillation is the widely used protocol for commercial purposes (Krishan and Narang, 2014). EOs are widely utilized in perfumery, food preservation, flavoring, and medicine (Bento et al., 2013).

The shelf life of meat products has been prolonged with the use of antioxidants in the meat industry (Lorenzo et al., 2018). EOs incorporated in animal diets increase meat antioxidant capacity, by preventing detrimental reactions resulting in lipid rancidity and significantly increasing consumer appeal owing to the prolonged storage life of meat (Zdanowska-Sasiadek*etal*., 2013). Herbs contain antioxidants that mitigate oxidation in products of animal origin and enhance antioxidant capacity of animal tissues (Abeyrathneet al., 2022), and delay the development of off-flavors by extending the shelf-life and relative measures of product stability. Shanoon et al., (2012) reported that EOs have antioxidative potential by retarding lipid peroxidation in chicken meat and enhancing the stimulation of endogenous enzymes.

Previous research indicates that refrigerated and long-term frozen storage of chicken meat increases lipid oxidation (Symeon et al., 2014). Turmeric prevents the accumulation of peroxide and prolongs the period of preserving foods (Ürüşan and Bölükbaşı, (2020). Supplementation of EOs has been successfully utilized in poultry; however, research is required to unravel its effects on the broiler meat of birds (Avila-Ramos et al., 2012).

The use of EOs in poultry nutrition can increase the antioxidant activity of meat without affecting its quality and composition. Supplements inclusions in diets have been proven to be an easy and affordable alternative to steadily incorporate natural antioxidants into phospholipid membranes to efficiently inhibit the oxidative reactions at their localized sites (Symeon et al., 2014).

Evidence of antagonistic effects by the addition of EOs in feeds is demonstrated by Avila-Ramos et al., (2012) who observed that the antioxidant capacity of oregano EO can be adversely affected in combination with acidulated soya bean oil in the diet. Hence, the inclusion of EOs in water offers better availability and efficiency. Despite the extensive research on EOs in poultry nutrition, there remains a gap in understanding the specific effects of EOs derived from spices on meat quality and oxidative stability in broilers. Given the diverse composition of spice-derived EOs and their potential synergistic interactions, investigating their impact on meat quality parameters and oxidative stability could unveil novel insights into optimizing broiler production practices and enhancing the nutritional value and shelf life of poultry products. Amidst the growing interest in natural additives for enhancing meat quality and extending shelf life, our study explores the innovative approach of incorporating EOs derived from spices into broiler water supplementation. Therefore, this study aims to assess the effect of oral supplementation with *Allium sativum, Curcuma longa, Zingiber officinale,* and *Cinnamomum zeylanicum* on the oxidative stability, cooking, and drip losses of broiler meat during frozen storage.

## Materials and Methods

### Ethical Approval

The institutional research and ethics committee of the Federal Polytechnic Ado Ekiti approved the experimental protocols of this study (AP/REC/2021/004), in conformity with NRC guidelines and the ARRIVE 2.0. The experiment was performed in line with guidelines of University Animal Scientific procedures [Animal (Scientific Procedures) Act 1986]. Adequate management practices were followed to reduce discomfort in the laboratory animals.

Fresh and mature turmeric (*Curcuma longa*), garlic (*Allium sativum*), cinnamon (*Cinnamomum zeylanicum*), and ginger (*Zingiber officinale*) were bought from a neighborhood spice market in Ado-Ekiti, Nigeria. The cinnamon bark, ginger rhizomes, turmeric rhizomes, and garlic cloves were manually diced to pieces, air-dried for 7 d at ambient temperature, and then blended into fine powder. As previously detailed by Dehariya et al. (2021), They were peeled, diced, and homogenized in water (40% w/v) and loaded into a cooking unit for hydrodistillation in a Clevenger-type apparatus. The extracted EO was dehydrated and stored in sealed glass vials below 18 °C. The EOs of garlic, cinnamon, ginger, and turmeric were extracted from their respective powders and were characterized and identified as garlic EO, turmeric EO, ginger EO, and cinnamon EO. Garlic EO contained allicin, diallyl trisulfide, diallyl disulfide, diallyl sulfide ajoene, s-allyl cysteine; cinnamon EO: cinnamaldehyde, eugenol, linalool, benzyl benzoate, cinnamyl acetate, β-caryophyllene; turmeric EO: curcumin, ar-turmerone, α-turmerone, β-turmerone, zingiberene, curcumene; ginger EO: gingerol, shogaol, zingerone, β-sesquiphellandrene, α-zingiberene, and β-bisabolene.

### Experimental Animals and Management

A 200-d-old chicks of arbor-acres broiler were procured from a local hatchery. The birds were allotted by weight to five treatments, each consisting of 10 replicates and four birds/replicate in a completely randomized design, designated as T1: no supplement, T2: garlic EO, T3: ginger EO, T4: turmeric EO, and T5: cinnamon EO. The birds were reared in cages and fed on a standard diet in line with the nutrient requirement at both phases of production. The spices’ EOs were supplemented (300 mL/L) into drinking water for the birds throughout the study. Feed were formulated as shown in Table 1 and freshwater were offered ad libitum. The hatchery-recommended vaccination program was followed and without other medication throughout the study.

**Table 1.** Gross composition and nutrient level of experimental diets

Ingredient	Starter diet	Finisher diet
Maize	58.5	66.5
Groundnut cake	9.8	8.3
Soybean meal	25	19.5
Fishmeal	3.1	2
Methionine	0.25	0.25
Lysine	0.25	0.25
Oyster shell	1.5	1.5
Limestone	1.1	1.2
Premix*	0.25	0.25
Salt	0.25	0.25
Total	100	**100**
Dry matter, %	85.848	85.546
Crude protein, %	22.992	18.575
ME, kcal/kg	3,031.27	3,086.43
Ether extract, %	3.9425	3.9305
Crude fiber, %	3.316	3.0325
Lysine, %	1.37755	1.17005
Methionine, %	0.60564	0.56059
Calcium, %	1.17455	1.15925
Phosphorus, %	0.31525	0.47845

ME, metabolizable energy (kcal/kg).

Premix* - Vitamin A–10,000,000 iu, Vitamin D3–2,000 iu, Vitamin E–40,000 mg, Vitamin K–2,000 mg, Vitamin B11,500 mg, Vitamin B2–4,000 mg, Vitamin B6–40,000 mg, Vitamin B12–20 mgr, Niacin–40,000 mg, Panthothenic–10,000 mg, Folic–1,000 mg, Biotin–100 mg, Choline Chloride–300,000 mg, Manganese–80,000 mg, Zinc–60,000 mg, Iron–40,000 mg, Copper–80,000 mg, Iodine–800 mg, Selenium–200 mg, Cobalt–300 mg, and Antioxidant–100,000 mg.

On the 50th d of the feed trial, three birds per replicate were randomly selected, and fasted for 12 h. Exsanguination was carried out with sharp knife by severing the jugular vein, carotid artery, and windpipe.

### Meat Quality

After dressing and evisceration, *Pectoralis major* (breast muscle) was weighed into plastic bag and inserted in water bath at 85 °C, before heat was applied. Samples were boiled until a defined internal temperature of 75 °C for 20 min, placed under running water for 15 min at ambient temperature. The meat samples were reweighed to calculate the percentage cooking loss (%) as follows:


$$\begin{array}{lll} Cooking\;loss\;\%=\; & \{Raw\;weight-Cooked\;weight\}\; & /Raw\;weight\;\;\;\times 100 \end{array}$$

{Raw weight}. In order to determine the drip loss of meat, meat samples of about 2.5 to 3.00 cm of thickness and approximate weight of 100 g of breast meat were inserted inside an expanded and closed bag and stored at 4 °C for 8 h. Drip loss was calculated as the loss in weight and expressed as a percentage relative to the initial weight.

### Oxidation Status in Pectoralis Major

After slaughter, dressing, and dissection into primal cuts, the breast meat was placed in sterile plastic containers, labeled per animal, and moved to the laboratory. Ten grams of breast meat samples in triplicates per animal were homogenized, centrifuged, and the supernatant obtained and designated. On day 0. The remaining breast meat samples were stored at 4 °C for weekly assessments. At days 7, 14, 21, and 28, an aliquot of 10 g samples in triplicate was removed post-thawing, homogenized, centrifuged, and the supernatant obtained, designated as respective days.

The homogenates were centrifuged (12,000 rpm for 45 min at 4 °C) in a centrifuge. The supernatant was assayed for protein, total antioxidant activity, lipid peroxidation, catalase, superoxide dismutase (SOD), and glutathione peroxidase (GPx) utilizing the method outlined in Jimoh et al., (2021).

### Statistical Analysis

The data were tested for normality and homogeneity of variances with levene’s test to ascertain the suitability of one-way ANOVA, before further analysis using IBM SPSS 20. Data obtained from the study were subjected to general linear model procedures of analysis of variance (*P* < 0.05). Mean differences were separated by the Duncan’s Multiple Range Test (DMRT) using IBM SPSS 20.

## Results

The effects of EO on the cooking and drip loss of breast meat originated from the carcass of broiler-fed supplemented EOs are shown in Table 2. There were significant (*P* < 0.05) differences in cooking loss among the five treatments. Cooking loss of *Pectoralis major* from birds on T1 was not significantly (*P* > 0.05) different from that of T4, and both were significantly higher than those of birds on T2, T3, and T5 which shared statistically similar values. Drip Loss ranged from 4.50% in meat of T1 birds to 1.50% in meat of T5 birds. *Pectoralis major* of T1 birds had the highest cooking loss similar to that of T4 birds while T5 had the lowest drip loss. There were significant (*P* < 0.05) differences in drip loss between T1 (4.5%) and T5 (3.00%). However, T2 (4.83%), T3 (4.17%), and T4 (3.00%) showed no significant (*P* > 0.05) differences in drip loss compared to control

**Table 2.** Physical properties of breast meat (*Pectoralis major*) from broilers fed essential oil extracts from different spices

	T1	T2	T3	T4	T5	SEM
Cooking loss (%)	34.17^a^	21.33^b^	22.83^b^	33.33^a^	24.17^b^	2.96
Drip loss (%)	4.50^a^	4.83^a^	4.17^a^	3.00^ab^	1.50^b^	0.42

a, b: Mean in the same row with different superscripts are significantly (*P* < 0.05) different. treatments (*n* = 3/group); SEM, standard error of mean; treatment 1, control (water: no additives) 0 ml/L; treatment 2, control + garlic essential oil at 30 mL/L; treatment 3, control + ginger essential oil at 30 mL/L; treatment 4, control + turmeric essential oil at 30 mL/L, and treatment 5, control + cinnamon essential oil at 30 mL/L.

**Figure 1.**
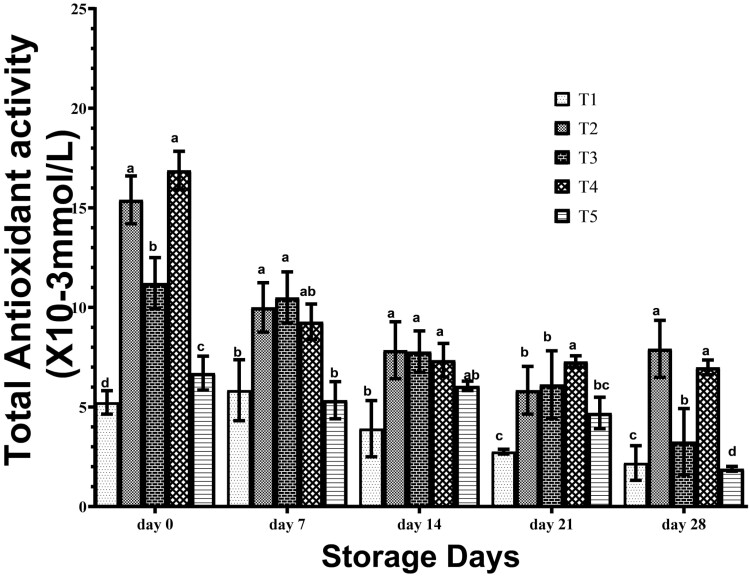
Antioxidant activity of frozen stored breast meat of broilers administered spices essential oils during storage. a, b, c, d, Mean in the same storage day with different superscripts are significantly (*P* < 0.05) different. Treatments (*n* = 3/group); SEM, standard error of mean. treatment 1, control (water: no additives) 0 mL/L, treatment 2, control + garlic essential oil at 30 mL/L, treatment 3, control + ginger essential oil at 30 mL/L, treatment 4, control + turmeric essential oil at 30 mL/L, and treatment 5, control + cinnamon essential oil at 30 mL/L.

The total antioxidant activity of frozen breast meat from birds administered with different EOs is shown in Figure 1. The total antioxidant activity of all the treatments decreased as the day of storage increased except T2 and T4 which were stable on 14th, 21st, and 28th day of storage. On day 0, values of total antioxidant activity in the meat of T2 birds and T4 birds were significantly (*P* < 0.05) higher than other treatments, while values obtained for T3 were higher than those obtained in meat of T5 birds and the significantly (*P* > 0.05) lowest values were obtained in meat of T1 birds. At 7th d, the total antioxidant activities of meat from T2 and T3 birds were significantly (*P* < 0.05) higher than those of T1 and T5 birds. At day 14, the total antioxidant activities of meat from T2, T3, and T4 birds were significantly (*P* < 0.05) higher than those on T1. At day 21, total antioxidant activities of breast meat from T4 birds were significantly higher than the T2 and T3 birds while the lowest values were obtained in T1 birds. At day 28, the total antioxidant activities of meat from T2 birds and T4 birds were significantly higher than the values obtained from T3, values of meat from T3 birds were significantly higher than the values recorded for T1 birds, with the lowest value obtained in meat from T5 birds. Total antioxidant activity declined in breast meat during storage, but the administration of EOs to birds fortified their antioxidant defense of the meat during storage, with birds administered garlic EO and turmeric EO having superior total antioxidant activity up to 28th d of frozen storage.

**Figure 2.**
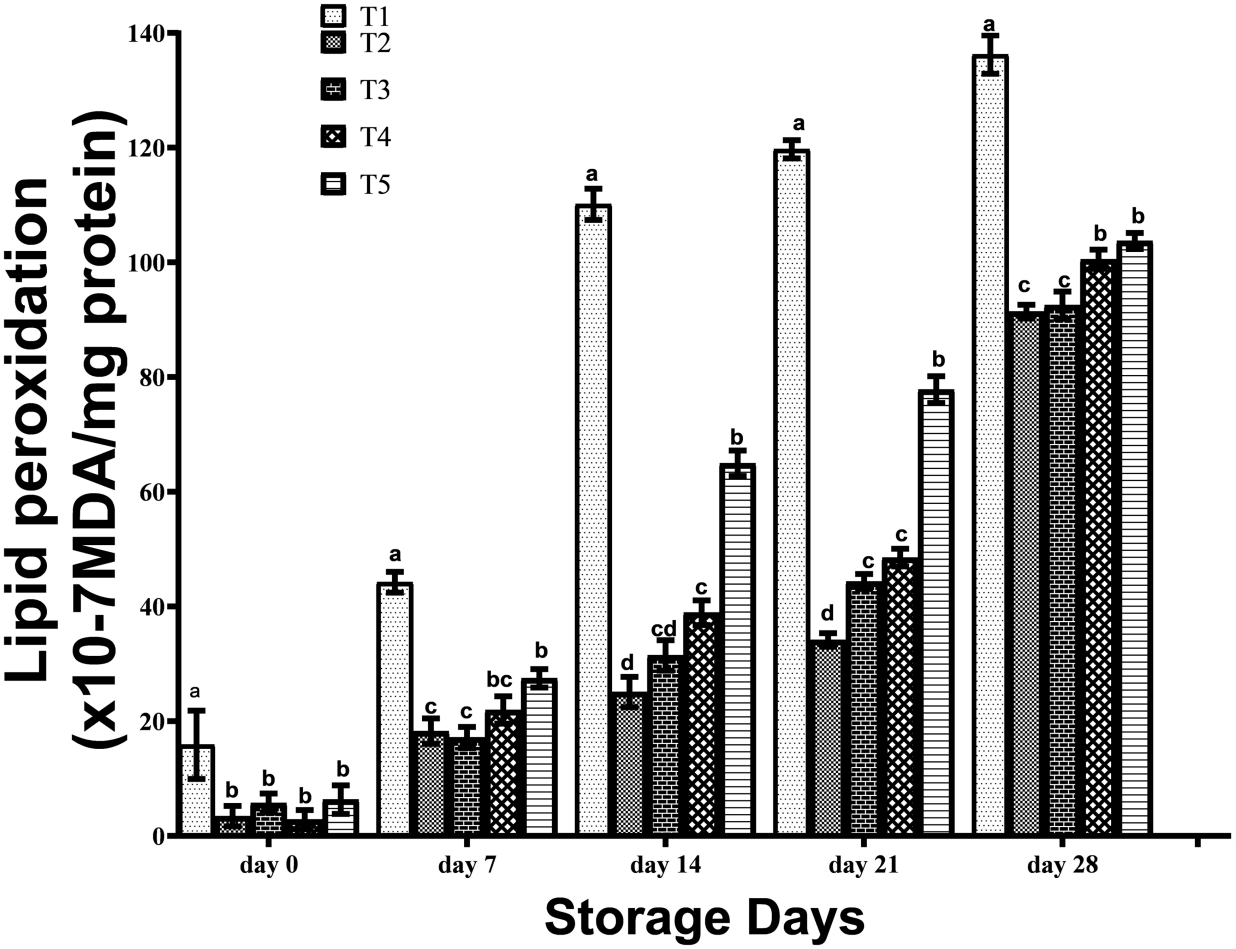
Lipid peroxidation of frozen stored breast meat of broilers administered spices essential oils during storage. a, b, c, d, Mean in the same storage day with different superscripts are significantly (*P* < 0.05) different. Treatments (*n* = 3/group); SEM, standard error of mean; treatment 1, control (water: no additives) 0 mL/L; treatment 2, control + garlic essential oil at 30 mL/L; treatment 3, control + ginger essential oil at 30 mL/L; treatment 4, control + turmeric essential oil at 30 mL/L, and treatment 5, control + cinnamon essential oil at 30 mL/L.

Effects of EO intake on lipid peroxidation values in frozen breast meat of broiler chickens are shown in Figure 2. On day 0, the rate of lipid peroxidation in breast meat of birds orally supplemented with EOs were statistically (*P* > 0.05) similar and significantly (*P* < 0.05) lower than those of control birds. At day 7, the lipid peroxidation in breast meat of T1 birds were also significantly (*P* < 0.05) highest and the least (*P* < 0.05) values were observed in those on T1 and T2 birds. On days 14 and 21, lipid peroxidation in breasts of T1 birds was significantly (*P* < 0.05) higher than other treatments, birds on T5 had higher (*P* < 0.05) values than T4 birds, with significantly (*P* < 0.05) least values obtained in T2 birds. On day 28, the rate of lipid peroxidation in breast meat of T1 birds was significantly (*P* < 0.05) higher than those of T4 and T5 birds which share statistically (*P* > 0.0) similar values, while birds that received T2 and T3 had the statistically (*P* < 0.05) lowest values. The trend of results shows that the ingestion of EOs by broiler chicken reduced the rate of lipid peroxidation in breast meat during storage with the best result obtained in T2 and T3 birds.

**Figure 3.**
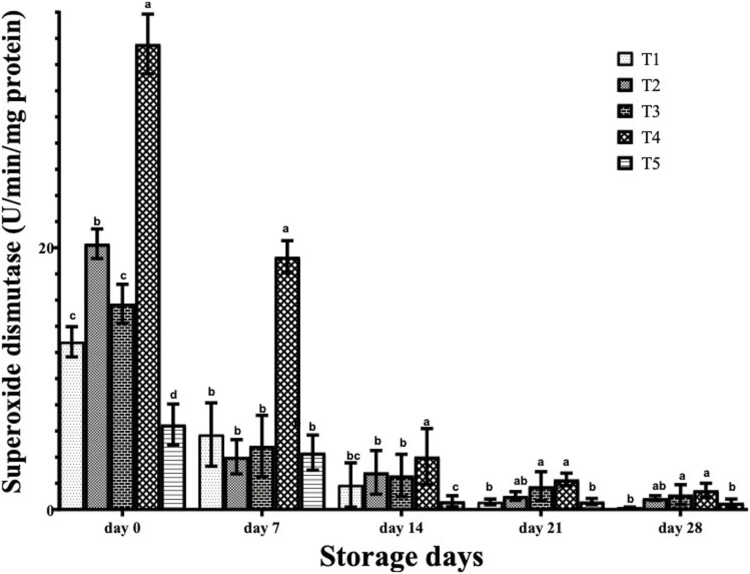
Superoxide dismutase of frozen stored breast meat of broilers administered spices essential oils during storage. a, b, c, d, Mean in the same storage day with different superscripts are significantly (*P* < 0.05) different. Treatments (*n* = 3/group); SEM: standard error of mean. Treatment 1, control (water: no additives) 0 mL/L; treatment 2, control + garlic essential oil at 30 mL/L; treatment 3, control + ginger essential oil at 30 mL/L; treatment 4, control + turmeric essential oil at 30 mL/L, and treatment 5, control + cinnamon essential oil at 30 mL/L.

Superoxide dismutase activity in frozen breast meat of birds that consumed spices EOs is shown in Figure 3. On day 0, SOD in the breast meat of birds on T4 was significantly (*P* < 0.05) higher than that of the other birds. The SOD values obtained in T1 and T3 birds were statistically (*P* > 0.05) similar and significantly (*P* < 0.05) higher than those of T5 birds. At day 7, SOD values of T4 birds were significantly (*P* < 0.05) higher than those of T1, T2, T3, and T5. At days 14, 21, and 28, the values of SOD have markedly decreased compared to days 0 and 7. However, T3 and T4 birds had significantly higher SOD activity in the breast meat compared to those on other treatments.

**Figure 4.**
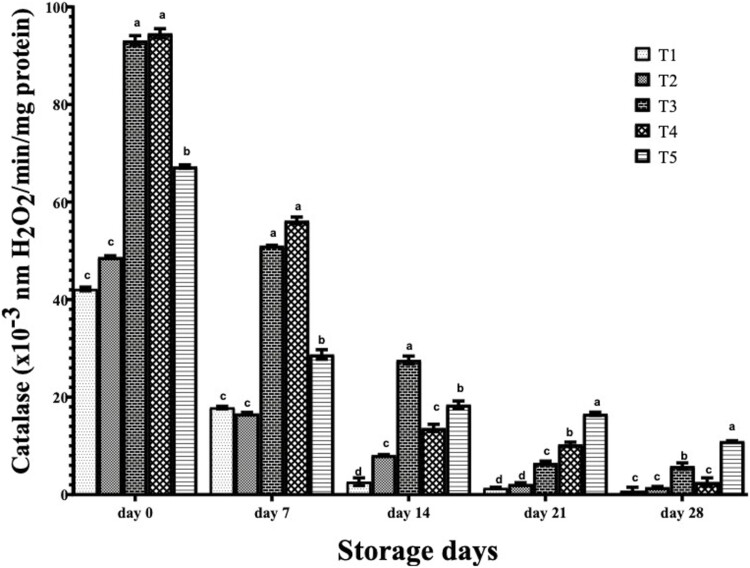
Catalase of breast meat of broilers administered spices essential oils during storage. a, b, c, d, Mean in the same storage day with different superscripts are significantly (*P* < 0.05) different. Treatments (*n* = 3/group); SEM, standard error of mean; treatment 1, control (water: no additives) 0 mL/L; treatment 2, control + garlic essential oil at 30 mL/L; treatment 3, control + ginger essential oil at 30 mL/L; treatment 4, control + turmeric essential oil at 30 mL/L, and treatment 5, control + cinnamon essential oil at 30 mL/L.

The catalase activity in frozen stored breast meat of birds administered with different EOs is shown in Figure 4. At days 0 and 7, the catalase activity of breast meat of T3 and T4 birds were statistically (*P* < 0.05) superior to those on T5, with the significantly (*P* < 0.05) lower values obtained in T1 and T2 birds. At day 14 catalase activity in breast meat of T3 birds were significantly (*P* < 0.05) higher than those of T5 birds, while T4 and T2 birds had significantly (*P* < 0.05) higher values than those on T1. At day 14, catalase activity in breast meat of T5 birds was significantly (*P* < 0.05) higher than those of T4 birds, while T3 birds had lower values than those of T4 and the significantly least values were obtained in T1 and T2 birds. At day 28, catalase activity in breast meat of birds on T1, T2, and T4 were statistically (*P* < 0.05) lower than other treatments, while T5 birds had significantly higher values than those on T3. The catalase activity of breast meat significantly declined from day 14, but was enhanced by EO consumption by the birds especially by T5 (cinnamon EO) up to 28 d. T3 (ginger EO) sustained catalase activity of the breast meat up to 14 d.

**Figure 5.**
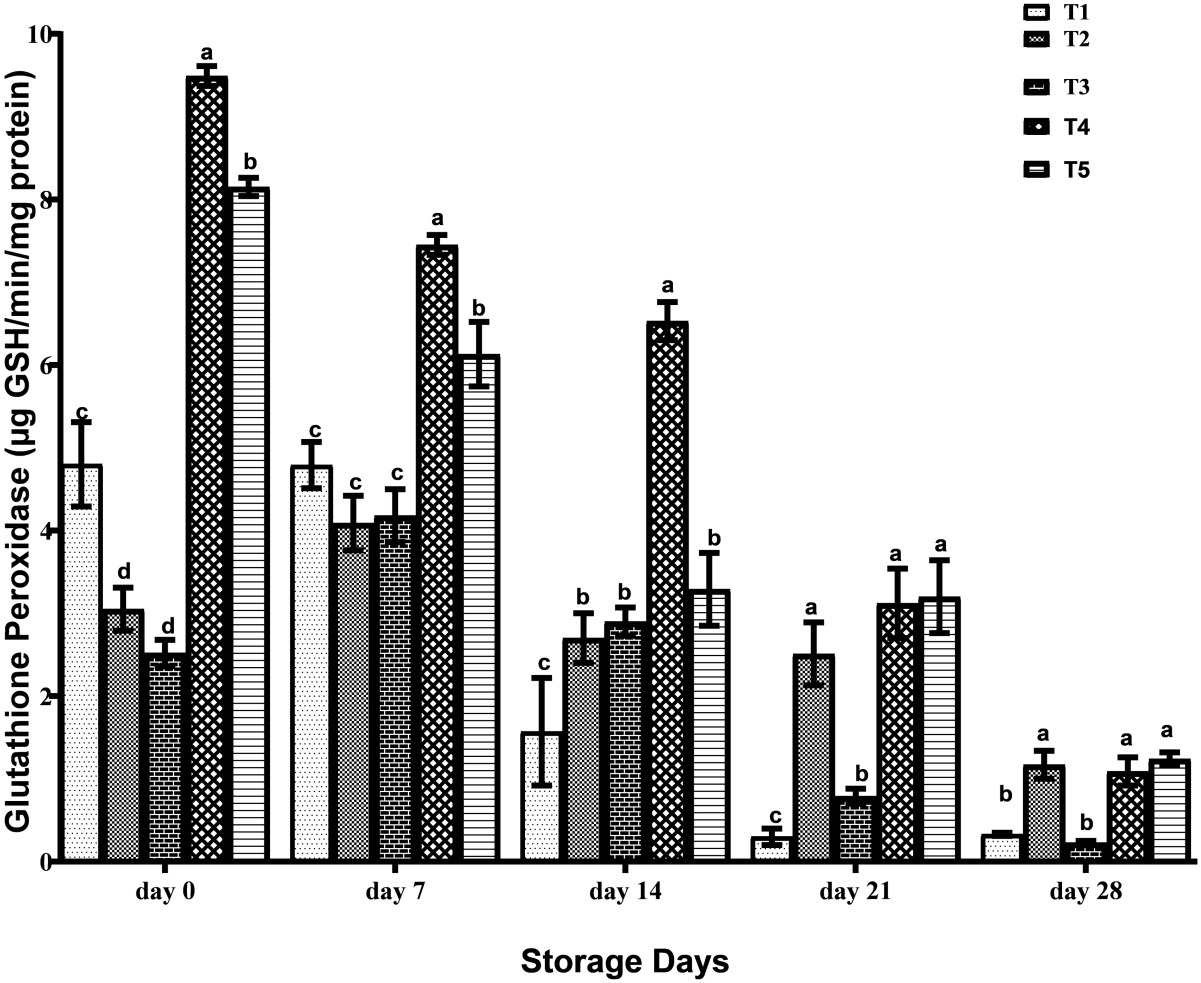
Glutathione peroxidase of breast meat of broilers administered spices essential oils during storage.a, b, c, d, mean in the same storage day with different superscripts are significantly (*P* < 0.05) different. Treatments (*n* = 3/group); SEM, standard error of mean; treatment 1, control (water: no additives) 0 mL/L; treatment 2, control + garlic essential oil at 30 mL/L; treatment 3, control + ginger essential oil at 30 mL/L; treatment 4, control + turmeric essential oil at 30 mL/L, and treatment 5: control + cinnamon essential oil at 30 mL/L.

The glutathione peroxidase activity in frozen stored breast meat of birds administered different EOs is shown in Figure 5. On day 0, the GPx activity of breast meat from birds on T5 was significantly (*P* < 0.05) lower than those on T4, while breast meat of T2 and T3 birds were significantly (*P* < 0.05) lower than those of T1 birds. On day 7, GPx activity of breast meat of T1, T2, and T3 birds were significantly (*P* < 0.05) lower than those of T5 birds, with the significantly (*P* < 0.05) highest value recorded in T4 birds. At day 14 GPx of breast meat of T4 birds was significantly (*P* < 0.05) higher than those on T2, T3, and T5, with the statistically (*P* > 0.05) least value obtained in T1 birds. On days 21 and 28, GPx activity of breast meat of T2, T4, and T5 birds were statistically (*P* > 0.05) similar and significantly (*P* < 0.05) higher than those of T1 and T3 birds. Supplementation of EOs to broiler chickens sustains GPx activity of breast meat up to 28 d, especially T2, T4, and T5 (garlic, turmeric, and cinnamon EO). The level of GPx in breast meat not administered EOs decreased drastically beyond 14 d.

## Discussion

Herbal feed additives have been reported to improve meat quality properties such as water-holding capacity, lightness, marbling, and oxidative stability (Puvaca et al., 2020). The considerable variation in physicochemical characteristics may be due to the proximate composition and of the EO extracts from *Allium sativum, Curcuma longa, Zingiber officinale,* and *Cinnamomum zeylanicum* Drip loss was the lowest in the meat of birds supplemented with cinnamon EO, while cooking loss was lower in breast meat of birds supplemented with garlic, ginger and cinnamon EO. Shrinkage of myofibrils and denaturation of sarcoplasmic proteins results in fluid loss. Low pH results in greater reduction in the WHC, but higher pH favors water retention and better WHC. Thus, it is suggested that meat pH influences the outcome of drip loss and cooking loss in meat quality. Similarly, Fellenberg and Speisky (2006) reported that the storage life of meat can be prolonged by the lasting effects of EO at postmortem.

Natural antioxidant supplements are utilized to prolong storage life and enhance consumer acceptability of chicken meat (Symeon et al., 2014). The antioxidant activities were obtained weekly to assess the effectiveness of different EO against the rancidity of *breast meat*. The storage caused a significant decrease in the antioxidant activity from 0 to 28 d of storage. It also revealed differences among treatments on antioxidant activity of *Pectoralis major* on each day of assessment. The constituents of the EO initiates the sequence of oxidative reaction (Augustyniak et al., 2010). Antioxidant activity of EO extracts is important because of the delay in oxidation, food preservation, and increases in their shelf life (Lorenzo et al., 2018).

These trends of results reveal that total antioxidant activity declines in breast meat during storage. However, the administration of EOs on birds fortified their antioxidant defense during storage, with birds administered with garlic EO and turmeric EO having superior total antioxidant activity of up to 28 of frozen storage. This finding is corroborated by the works of Al-Hijazeen et al. (2016) who reported the superiority of antioxidant activity of meat from birds on oregano EO, such that it is a better preservative for chicken meat than synthetic antioxidant. The result of this study is corroborated by claims that antioxidant activities decreased by storage of meat, whereas the malondialdehyde (MDA) values increased by Avila-Ramos et al., (2012).

However, birds on turmeric EO had higher SOD activity in the chicken meat compared to those on other treatments. This agreed with Ürüşan and Bölükbaşı, (2020) who reported that turmeric dietary supplementation exerted postmortem effects by increasing meat shelf life and reducing lipid oxidation rates owing to the quantity of the active ingredients of turmeric in tissues.

Similarly, administration of EO has been revealed to protect against oxidation in meat and meat products (Krishnaraju et al., 2009). In agreement with this study is the claim that EOs positively impact antioxidant enzymes and changes in the enzymes are due to the active compounds in the EO extracts (Miguel, 2010). Thus, the supplementation of EOs in feed may markedly improve the welfare and storage life of chicken meat (Decker and Park, 2010).

Chicken meat is rich in PUFA, making it prone to oxidative deterioration (lipid oxidation) during storage, via the production of several volatile molecules responsible for rancidity and off-flavor development in meat (Al-Hijazeen et al. 2016). Lipid peroxidation defines the magnitude of rancidity occurrence resulting from auto-oxidation of meat. Lipid oxidation is a critical meat quality parameter because it is primarily responsible for producing many off-odor and rancid flavors in the meat during storage, and also influences protein oxidation that leads to meat discoloration (Al-Hijazeen et al. 2016). The rate of lipid peroxidation increased as storage days increased. However, the EOs consumption reduced the peroxidation rates markedly in comparison with those on control. This could be due to the active ingredients in the EOs, which have been reported to prevent peroxidation in lipids (Kamboh and Zho 2013). The bioactive compounds in cinnamon oil have antioxidant properties, which react with lipid and hydroxyl radicals to convert into stable products while being reduced (Symeon et al., 2014). Also, researchers have reported a decreased lipid peroxidation when turmeric was incorporated into the diet (Botsoglou et al. 2002; Daneshyar, 2012; Hosseini-Vashan et al., 2012). This could be through the absorption of the compounds into the tissues, where they mitigate reactions leading to lipid oxidation (Azevedo et al., 2021).

The rate of lipid peroxidation increased as storage days increased. However, the EOs consumption reduced the peroxidation rates markedly compared with meat originating from control broilers. The result of this study is corroborated by claims that MDA values of breast meat increased during storage (Gharejanloo et al., 2017). Also, Gharejanloo et al. (2017) reported the suitability of thyme and rosemary EOs to maintain oxidative stability of breast meat over 30 d of preservation at −20 °C

This study agrees with Akbarian et al. (2014) who demonstrated that inclusion of turmeric and oregano oils in the diet reduced malondialdehyde levels in chicken meat. Contrary to the result of this study, 750 mL/1000L EO mixture to water consumed by broilers under heat stress did not affect the TBARS values in the breast meat compared to the control group (Tekce et al., 2020). Heat stress condition poses oxidative shock on animal physiology generally, thus increasing the antioxidant demand to control oxidation processes. In the current study at 3 mL/100 mL, oxygen species generated post-slaughter were combated by the antioxidants fortified in the muscles before slaughter. Hence EO was able to combat lipid peroxidation in meat from birds on EO-based treatments compared with the control. This is supported by reports, which affirmed that enriching animal feeds with EO extracts increases the antioxidant potential in meat, which ameliorates adverse reactions resulting in fat rancidity and can appeal to consumers owing to the prolonged storage shelf life of meat (Zdanowska-Sasiadek et al., 2013). Similarly, addition of turmeric powder to broiler ratio decreased the lipid oxidation rate of the thigh, as an effect of curcumin (Ürüşan and Bölükbaşı, (2020). Numerous researchers have reported that curcumin (the active ingredient of *Curcuma longa)* has an antioxidant activity comparable with that of Vitamin C and E (Ürüşan and Bölükbaşı, (2020). Comparatively, garlic and ginger EO administration to broiler chickens lowered lipid peroxidation in breast meat over 28 d compared to those administered other spices EOs. This is supported by claims that different EOs have varying capacities to influence meat oxidative stability. This as demonstrated by Anjum et al. (2004), who reported that oxidized soybean oil led to greater accumulation of MDA in meat than those of crude soybean oil. Similarly, Fratianni et al. (2010) reported that thyme EO improves the storage life of fresh breast meat stored at 4 °C by preventing lipid peroxidation and maintaining the meat quality beyond 2 wk of storage. Azevedo et al., (2021) reported that lemongrass and pedestrian tea EOs are potent antioxidant supplements in broiler nutrition capable of improving the oxidative stability of meat during storage.

Avila-Ramos et al., (2012) also reported a higher concentration of MDA in the meat from birds administered acidulated soybean oil compared with meat samples from birds on crude soybean oil. Gharejanloo et al. (2017) reported that supplementing rosemary and thyme EOs was successful in reducing MDA and had a higher antioxidant effect on the chicken meat.

In contrast, Symeon et al. (2014) claimed that the incorporation of cinnamon oil into the broiler’s ratio had no influence on lipid oxidation of refrigerated and long-term frozen breast meat.

In this study, drip and cooking losses recorded in meat from birds on cinnamon EO, shows enhanced physicochemical properties of the meat. The trend of lowered lipid peroxidation rate and enhanced antioxidant profile conferred on the breast meat of birds administered with EOs promotes the storage life of the meat. Other authors have reported that antioxidant fortification of chicken diets is an excellent method of reducing saturated fatty acids and enhancing PUFA in chicken meat, and could directly impact the physicochemical and shelf-life of meat (Sierżant et al., 2018). Also, Rimini et al. (2014) reported that EOs from oranges or thyme are natural preservative agents of chicken meat, by preventing lipid peroxidation in meat without affecting its quality. Dietary antioxidant supplements allow assimilation into subcellular membranes to effectively inhibit the oxidation reactions at their localized sites and elucidate improved meat quality owing to amelioration of lipid peroxidation and muscle discoloration (Symeon et al., 2014).

## Conclusion

The study reveals that the EOs (*Allium sativum, Curcuma longa Zingiber officinale,* and *Cinnamomum zeylanicum*) are valuable supplements for broiler chickens and potent in enhancing meat quality and prolongs the shelf life of the meat for 28 d by regulating oxidative stability during frozen storage. The consumption of different EOs added to the drinking water of broilers exerted different mechanisms in broiler meat during frozen storage. Cinnamon EO reduced drip and cooking losses, and maintained GPx and catalase activity also sustained meat oxidation over 28 d, Turmeric EO sustained GPx for 14 d and SOD for 7 d while ginger EO reduced cooking loss and sustained catalase activity in meat for up to 14 d.
